# Energy expenditure associated with posture transitions in preschool children

**DOI:** 10.1371/journal.pone.0215169

**Published:** 2019-04-15

**Authors:** Katherine L. Downing, Xanne Janssen, Dylan P. Cliff, Anthony D. Okely, John J. Reilly

**Affiliations:** 1 Institute for Physical Activity and Nutrition (IPAN), School of Exercise and Nutrition Sciences, Deakin University, Geelong, Australia; 2 School of Psychological Sciences and Health, University of Strathclyde, Glasgow, Scotland; 3 Early Start, School of Education, Illawarra Health and Medical Research Institute, Faculty of Social Sciences, University of Wollongong, Wollongong, Australia; California State University San Marcos, UNITED STATES

## Abstract

**Background:**

Despite growing scientific interest in the benefits of breaking up sedentary time with intermittent standing or walking, few studies have investigated the energy cost of posture transitions. This study aimed to determine whether posture transitions are associated with increased energy expenditure in preschool children.

**Methods:**

Forty children (mean age 5.3 ± 1.0y) completed a ~150-min room calorimeter protocol involving sedentary, light, and moderate- to vigorous-intensity activities. This study utilised data from ~65-min of the protocol, during which children were undertaking sedentary behaviours (TV viewing, drawing/colouring in, and playing with toys on the floor). Posture was coded as sit/lie, stand, walk, or other using direct observation; posture transitions were classified as sit/lie to stand/walk, sit/lie to other, stand/walk to other, or vice versa. Energy expenditure was calculated using the Weir equation and used to calculate individualised MET and activity energy expenditure (AEE) values. Spearman’s rank correlations were used to compare the number of posture transitions, in the individual activities separately and combined, with corresponding MET and AEE values. Participants were divided into tertiles based on the number of posture transitions; MET and AEE values of children in the lowest and highest tertiles of posture transitions were compared using unpaired t-tests. Effect sizes (Cohen’s *d*) were calculated.

**Results:**

There was a positive correlation between the total number of posture transitions and average METs (*r*_*s*_ = 0.42, p = 0.02) and AEE (*r*_*s*_ = 0.43, p = 0.02). MET differences between the lowest and highest tertiles of posture transitions resulted in a small effect size for playing with toys (*d =* 0.27), and moderate effect sizes for TV viewing, drawing and all three activities combined (*d =* 0.61, 0.50 and 0.64 respectively). Similar results were found for AEE.

**Conclusions:**

Results from this study showed that variation in posture transitions may be associated with variation in energy expenditure in preschool children. The findings suggest that the concept that variation in posture transitions may have meaningful biological or health effects in early childhood is worth investigating further.

## Introduction

Sedentary behaviour, defined as any activity undertaken in a sitting or lying posture and requiring fewer than 1.5 metabolic equivalents (METs) [1], has an impact on several major health outcomes in adults. High levels of sedentary behaviour are associated with an increased risk of all-cause mortality, cardiovascular disease, cancer, and type 2 diabetes, largely independent of time spent in moderate- to vigorous-intensity physical activity (MVPA) [2]. Breaking up prolonged bouts of sedentary time has been shown to be associated with more favourable cardiometabolic profiles in adults [3, 4], with similar evidence emerging in children [5–8]. Although the direct mechanisms are still largely unknown, these associations are likely due to distinct and important physiological differences in skeletal muscle metabolism and energy expenditure that exist between sitting and standing still [9].

Despite the growing scientific interest in the benefits of breaking up sedentary time with intermittent standing or walking, few studies have investigated the actual energy cost of changes in posture (e.g., sit/stand transitions). Understanding the relationship between posture transitions and energy expenditure might better explain individual differences in overall activity energy expenditure, which has important implications for energy balance. The recent sedentary behaviour *Terminology Consensus Project* called for more research on the physiological impact of posture transitions [1]. Judice et al. [10] examined the metabolic/energy cost for sitting, standing and sit/stand transitions in a sample of 50 adults: continuous standing had a metabolic cost of 0.07 kcal min^-1^ more than continuous sitting (a rise in metabolic rate of around 5–8%). However, a single sit/stand/sit transition had a metabolic cost of 0.32 kcal min^-1^ more than continuous sitting (a rise of around 35%). These findings may be particularly relevant for young children given there is evidence of more frequent posture transitions in pre-schoolers when compared to older children/adolescents [11] and adults [12]. To our knowledge no studies have investigated the energy cost of posture transitions in preschool-aged children. This study therefore aimed to determine whether posture transitions are associated with increased energy expenditure in preschool children.

## Methods

### Recruitment and participants

The present study involved secondary analyses of data from a larger study that aimed to validate various objective measures of free-living energy expenditure and physical activity in young children against a criterion measure (energy expenditure using whole room calorimetry [WRC]) [13]. In 2011, 40 healthy 4- to 6-year-old children were recruited from childcare centres (pre-schools, long-day and family-day care) in the Illawarra region of New South Wales, Australia. Exclusion criteria included the child having a disease known to influence their energy balance (e.g., hypothyroidism), a physical disability, or claustrophobia; no children were excluded based on these criteria. The study was approved by the University of Wollongong/South Eastern Sydney and Illawarra Area Health Service Human Research Ethics Committee. All participating parents provided written informed consent and children provided verbal assent.

### Measures and data management

Children and their parents had a familiarisation visit at the university before the measurement [14, 15]. During the measurement visit (which occurred within a week of the familiarisation visit), children completed a 150-minute activity protocol including age-appropriate sedentary behaviours and physical activities within a WRC. Children ate a light, standardized breakfast 1.5 hours before entering the WRC, which was shown to have a minimal impact on their energy expenditure [16]. The present study utilised only data from the first approximately 65 minutes of the activity protocol, during which children were undertaking sedentary behaviours [13]. The remainder of the protocol (involving physical activities) was excluded because: a) the energy expenditure of any posture transitions could not be differentiated from the energy expenditure of the activities themselves, and; b) there would be minimal posture transitions during these activities. The duration and order of the activities was pre-set and the same for each child (see Table 1). Children completed one activity before moving on to the next. Children were instructed to complete the activity while in a seated position, as they would do in a free-living situation; however, they were not specifically instructed to sit still (i.e., they had a degree of freedom over how they completed each activity). Talking on the telephone and reading were excluded from the present study as they were <10 minutes duration, meaning that stable measures of energy expenditure could not be calculated [17], resulting in approximately 60 minutes of the protocol remaining for analyses.

**Table 1:** Whole room calorimeter protocol.

Activity	Time (min)
Watching TV–sitting on a beanbag	30
Talking on telephone with parents–sitting	2
Reading books with a cassette/CD–sitting	5
Drawing/colouring in–sitting	10
Playing with toys, blocks (Lego), dolls, puzzles, games–sitting on floor	20

Children were filmed whilst completing the protocol, with activity start/end times and breaks between activities recorded. Video footage was coded by one observer using Vitessa (Version 0.1, University of Leuven, Belgium), which generated a time stamp every time a change in posture was coded. Every second following a given time stamp was coded as being at the same posture as that occurring at the point of the time stamp itself. Each second was coded in this way until a change in posture was indicated, resulting in second-by-second coding. Children's postures were classified as sit/lie, stand, step or ‘other’ (i.e., postures that did not fit in the other categories, such as kneeling on one knee, crawling, or hanging over the edge of a chair while leaning on a table [18]). For the purposes of the present study, a change in posture was classified as sit/lie to stand/walk, sit/lie to other, stand/walk to other, or vice versa. The number of posture transitions whilst watching TV, drawing/colouring in, and playing with toys were counted for each child. In addition, the number of posture transitions in each of these three activities were summed.

Oxygen consumption (VO_2_) and carbon dioxide production (VCO_2_) were measured continuously (paramagnetic O_2_ and infrared CO_2_ analyzers, Sable System Inc, Las Vegas USA) and corrected to standard temperature, pressure and humidity in the room calorimeter (3 m × 2.1 m × 2.1 m). Technical procedures have been previously described in detail [16]. As per Schoffelen et al. [17], chamber air was sampled every two minutes and rates of O_2_ consumption and CO_2_ production were calculated from in- and outflow. Rates of O_2_ consumption and CO_2_ production were then averaged over 10 minutes to produce stable measures of energy expenditure [16], and rates of energy expenditure were calculated using the Weir equation [19]. Individualised MET values were calculated by dividing measured energy expenditure for each child by their predicted basal metabolic rate (BMR). BMR was individually estimated for each child using equations developed by Schofield et al. [20] in 3- to 10-year-old children. Activity energy expenditure (AEE) was calculated by deducting BMR from measured energy expenditure. The average MET and AEE values were identified for each child whilst watching TV, drawing/colouring in, and playing with toys. MET and AEE values were also averaged across all three activities.

### Statistical analyses

Analyses were conducted in Stata 15.0 (StataCorp, Texas, USA). Descriptive statistics were used to characterise the sample. Initially, Spearman’s rank correlations were used to investigate if the number of posture transitions while: 1) watching TV; 2) drawing/colouring in, and; 3) playing with toys, were correlated with the corresponding MET and AEE values during those activities. The correlation between the total number of posture transitions across the three activities combined and the average MET and AEE values were also examined. Participants were divided into tertiles based on the number of posture transitions in each activity and for the combined activities. The energy expenditure of children in the lowest and highest tertiles of posture transitions were compared using unpaired t-tests. Given the small sample size, effect sizes (Cohen’s *d*) were also calculated; values of 0.20 represent small, 0.50 moderate, and ≥0.80 large effect sizes [21]. The power of the current study was fixed by the sample size recruited to the original study [13].

## Results

Of the 40 children who completed the WRC protocol, two had missing data due to calorimeter malfunction. Of the remaining 38 children, 32 (84.2%), 36 (94.7%), and 35 (92.1%) had valid energy expenditure data for TV viewing, drawing and playing with toys, respectively. Thirty-one children had valid data for the combined analyses. Descriptive characteristics of the current sample (n = 36) are presented in Table 2. The mean (SD) measurement time for the three activities was 56.6 (7.3) minutes.

**Table 2:** Participant characteristics; mean (SD) unless otherwise noted.

Child characteristic	Total sample (n = 36)
Age, years	5.3 (1.0)
Sex (% male)	55.6%
Height, cm	112.9 (8.4)
Weight, kg	20.6 (3.8)
BMI, kg/m^2^	16.0 (1.5)
BMI z-score*	0.5 (1.0)

Notes:

* Age- and sex- specific z-scores calculated based on WHO Child Growth Standards

Abbreviations: BMI = body mass index; SD = standard deviation

Spearman’s rank correlations between the number of posture transitions and energy expenditure are shown in Table 3. There were no significant correlations between the number posture transitions and either METs or AEE for each of the individual activities (i.e., TV viewing, drawing and playing with toys). However, when all three activities were combined, there was a significant, positive correlation between the total number of posture transitions and average METs (*r*_*s*_ *=* 0.42, *p =* 0.02) and AEE (*r*_*s*_ *=* 0.43, *p =* 0.02).

**Table 3:** Spearman’s rank correlations between number of posture transitions and energy expenditure (METs and AEE).

	METs		AEE	
Activity	Spearman’s rho	*p*	Spearman’s rho	*p*
TV viewing	0.28	0.12	0.23	0.20
Drawing	0.16	0.35	0.19	0.28
Playing with toys	0.26	0.13	0.28	0.10
Combined	0.42	0.02*	0.43	0.02*

Notes:

* Indicates statistical significance (p<0.05)

Abbreviations: AEE = activity energy expenditure; METs = metabolic equivalents

Table 4 shows the mean number of posture transitions in each of the individual activities (and combined) for the whole sample and for the lowest, middle and highest tertiles. The fewest posture transitions were observed during TV viewing, while the most transitions were observed during playing with toys. There were no statistically significant differences in the number of posture transitions between boys and girls (data not shown).

**Table 4:** Number of posture transitions during activities for total sample and in lowest, middle and highest tertiles.

	Total sample		Lowest tertile for posture transitions		Middle tertile for posture transitions		Highest tertile for posture transitions	
Activity	N	Mean (SD) posture transitions	N	Mean (SD) posture transitions	N	Mean (SD) posture transitions	N	Mean (SD) posture transitions
TV viewing	32	5.1 (7.2)	13	0.0 (0.0)	10	3.3 (1.5)	9	14.3 (7.5)
Drawing	36	6.4 (6.0)	13	1.1 (1.3)	11	5.5 (1.5)	12	13.0 (5.4)
Playing with toys	35	15.5 (17.8)	12	3.3 (1.9)	12	11.6 (3.3)	11	33.2 (22.8)
Combined	31	27.4 (23.1)	13	10.8 (4.9)	8	22.0 (3.2)	10	53.4 (23.9)

Abbreviations: SD = standard deviation

The mean differences in METs for the lowest and highest tertiles of posture transitions in each of the individual activities (and combined) are shown in Table 5. Although not statistically significant, the differences in METs observed for TV viewing and drawing resulted in moderate effect sizes (*d =* 0.61 and 0.50 respectively). When the three activities were examined in combination, the differences in METs also resulted in a moderate effect size (*d* = 0.64). Similar results were found for AEE (Table 6).

**Table 5:** Comparison of mean METs in lowest and highest tertiles for posture transitions.

Activity	Mean (SD) METs lowest tertile for posture transitions	Mean (SD) METs highest tertile for posture transitions	Mean (95% CI) difference in METs	*p*	Cohen’s *d*
TV viewing	1.21 (0.13)	1.40 (0.46)	0.19 (-0.09, 0.46)	0.18	0.61
Drawing	1.44 (0.31)	1.61 (0.36)	0.17 (-0.11, 0.44)	0.22	0.50
Playing with toys	1.36 (0.22)	1.42 (0.26)	0.07 (-0.14, 0.28)	0.52	0.27
Combined	1.32 (0.18)	1.46 (0.25)	0.14 (-0.05, 0.32)	0.14	0.64

Abbreviations: CI = confidence interval; diff = difference; METs = metabolic equivalents; SD = standard deviation

**Table 6:** Comparison of mean AEE (kcal/min/kg) in lowest and highest tertiles for posture transitions.

Activity	Mean (SD) AEE lowest tertile for posture transitions	Mean (SD) AEE highest tertile for posture transitions	Mean (95% CI) diff in AEE	*p*	Cohen’s *d*
TV viewing	0.007 (0.004)	0.013 (0.016)	0.006 (-0.003, 0.016)	0.19	0.59
Drawing	0.013 (0.009)	0.020 (0.013)	0.006 (-0.003, 0.015)	0.18	0.56
Playing with toys	0.011 (0.007)	0.014 (0.009)	0.002 (-0.004, 0.009)	0.47	0.31
Total	0.010 (0.006)	0.015 (0.009)	0.005 (-0.002, 0.011)	0.15	0.64

Abbreviations: AEE = activity energy expenditure; CI = confidence interval; diff = difference; SD = standard deviation

## Discussion

Despite growing interest in the metabolic health effects of breaking up sitting, few studies have examined the energy cost of changes in posture and none have done so in children aged 4–6 years. Emerging evidence in adults suggests that sit/stand transitions have a significantly higher energy cost than sitting still [10]. Findings from the present study suggest that children who have more frequent changes in posture during typical sedentary behaviours may expend more energy than those who have less frequent posture changes. With the exception of time spent playing with toys, the effect sizes for the differences in energy expenditure between the lowest and highest tertiles of posture transitions in individual activities (i.e., watching TV and drawing), and for the combined activities, were moderate. Additionally, there was a statistically significant positive correlation between the total number of posture transitions during sedentary time and energy expenditure, suggesting that the notion that variations in posture transitions produces meaningful variation in energy expenditure in young children is worth investigating further.

Although the magnitude of the associations observed in the present study might be interpreted as modest in the short-term, they may be clinically significant when the large amount of time young children spend sedentary per day, and when the cumulative influence over days, weeks, and months, are considered. The long-term cumulative influence of small increases in energy expenditure may be important for overall energy balance, and subsequently body fatness, in the longer-term. For example, in a study comparing posture allocation in lean and mildly obese participants, Levine et al. [22] found that obese individuals sat for around two hours more per day than lean individuals. Posture allocation remained the same when the obese individuals lost weight and when lean individuals gained weight, suggesting that movement (and posture transitions) may be biologically determined. The authors concluded that if the obese individuals adopted the behaviours of their lean counterparts (i.e., sat less), they could theoretically expend an additional 350kcal per day [22]. In the current study, the small difference in AEE of 0.005 kcal/min/kg between children with high vs low posture transitions may be meaningful if sustained and accumulated over days, weeks, months and years. Preschool children spend on average around 10 hours per day sedentary [23]. The difference in AEE across 10 hours of sedentary time, for a child weighing 20kg (mean weight in the current study), would result in 21,900kcal per year (almost 3kg/year). This suggests the difference may be biologically and clinically meaningful.

It is important to consider that the present study was a conservative test of the influence of posture transitions on energy expenditure; effects were likely constrained by the duration of the protocol. In addition, the number and nature of the transitions were limited by the setting of the WRC and the specific guidance which children were asked to follow, i.e., they were asked to only carry out the activities instructed for the durations instructed. In free-living conditions, a transition may be associated with more movement than was possible or allowed in the WRC. Participating children in the present study were also not preselected for any tendency to have high or low rates of posture transitions. However, despite the constraints of the sample, the protocol and the WRC, there was substantial variation in the number of transitions, ranging from 11 posture transitions per hour in the lowest tertile to 53 posture transitions per hour in the highest tertile, as children had some degree of freedom over how they completed each activity. This suggests that there may be inherent between-child differences in the tendency to transition between postures, which may potentially be related to inherent predispositions to fidgeting/restlessness.

Levine et al. [24] examined the changes in energy expenditure with fidgeting-like activities in a sample of adults; the energy expenditure of these activities (e.g., hand and foot tapping, arm and leg swinging) while seated was significantly greater than the energy expenditure while sitting motionless. Building on this, Koepp et al. [25] showed that chairs and devices that promote fidgeting increase energy expenditure by ∼20–30%. Self-reported fidgeting has also been shown to be associated with reduced all-cause mortality risk in adults, suggesting that fidgeting may reduce the risk of all-cause mortality associated with excessive sitting time [26]. In the present study, the number of posture transitions may be a crude, but convenient, proxy for underlying fidgeting tendencies. Of note, we observed a greater number of posture transitions during drawing (average of six transitions in ~10 min) and playing with toys (average of 16 transitions in ~10 min) compared to watching TV (average of five transitions in ~30 min). Potentially children move more (i.e., fidget more) when engaged in interactive activities like drawing or playing with toys compared to more passive activities such as watching TV programs. This may be important from a public health perspective and provides further evidence to suggest that children should be encouraged to partake in more interactive than passive sedentary behaviours. Evidence in older children supports this notion, suggesting that different types of sedentary behaviour may have different impacts on metabolic health [27].

Limitations of the current study must be acknowledged. Firstly, the study was not powered in relation to the hypothesis being tested, and secondly the study participants were not preselected for their tendency to fidget or not fidget. The findings are therefore conservative, and more substantial influences of variation in posture transitions on variation in energy expenditure might be observed in free-living conditions. Despite the small sample size, a significant positive correlation was observed between the number of posture transitions and energy expenditure. Due to the calorimeter sampling frequency and the time lag that exists when measuring energy expenditure in large volumes, it is difficult to identify short-term changes in energy expenditure using the WRC [17]. It is therefore possible that some energy expenditure as a result of posture transitions may have been misclassified between short, individual activities. This might have contributed to the smaller correlations for the individual activities, but may have been less influential when activities were combined and the correlation with energy expenditure was tested over a longer duration. Because of the WRC time lag, we were unable to examine the influence of different types of posture transitions (e.g., sit to stand, stand to other) on energy expenditure. Additionally, we were unable to separate sit and lie to examine whether posture transitions from a sitting position have different associations with energy expenditure compared to posture transitions from a lying position. Future studies should aim to examine the potentially different associations of different types of posture transitions on energy expenditure. Finally, WRC protocols for young children need to be less restrictive than in adult calorimetry [16], e.g., prolonged fasting prior to the measurements is not feasible or ethical, but protocols are reliable and valid and sufficiently robust for hypothesis-testing studies of the kind reported here [15, 16].

## Conclusions

Findings from this study suggest that posture transitions may be associated with increased energy expenditure in preschool children. Despite the inherent limitations, the findings are encouraging and provide preliminary evidence to suggest that the concept that variation in posture transitions may have meaningful biological or health effects in early childhood is worth investigating further.

## Supporting information

S1 Appendix Data file. (DTA)
